# Amino Acid Residue-Specific Ramachandran Distributions Derived from a Simple Mean Field Potential

**DOI:** 10.1021/acsphyschemau.4c00064

**Published:** 2024-10-21

**Authors:** Brian Andrews

**Affiliations:** Department of Physics, Bryn Mawr College, Bryn Mawr, Pennsylvania 19010, United States

**Keywords:** amino acid residue, conformational preferences, Ramachandran distribution, pPII, unfolded state, mean field

## Abstract

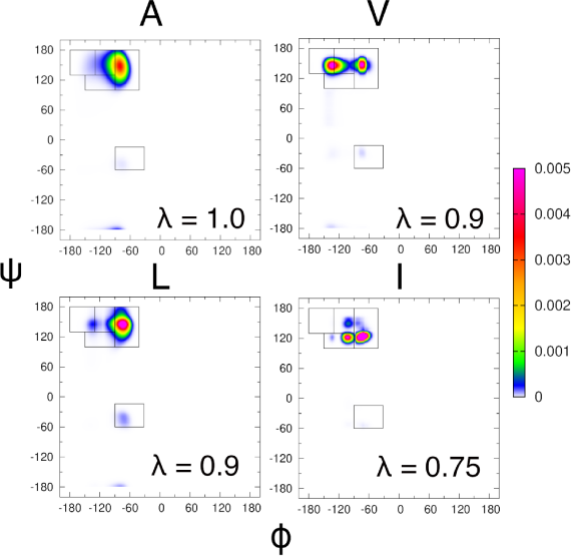

Protein dynamics in the unfolded state, in the context of early stage protein folding or intrinsically disordered proteins (IDPs), is not well understood. The discovery of IDPs, and their sequence-dependent dynamics, has led to many computational and experimental investigations regarding the conformational preferences of short oligopeptides and individual amino acid residues in the unfolded state. As proteins consist of sequences of amino acid residues, characterizing the intrinsic conformational preferences of the individual residues in the unfolded state is crucial for understanding the emergent conformations of peptides and proteins. While advances have been made in understanding conformational preferences, the atomistic mechanisms driving these preferences remain unresolved. In this work, we show that the distributions of atomic overlaps between backbone and side chain atoms in Ramachandran space are unique for amino acid residue mimetic structures alanine, valine, leucine, and isoleucine in Ramachandran space indicating unique intrapeptide energy landscapes for each residue. We then construct a mean field potential consisting of only an empirical peptide backbone–water and average intrapeptide Lennard-Jones contributions to explore their influence on the conformational preferences. With this fairly simple model, we were able to produce Ramachandran distributions that qualitatively agree with previously reported experimental and computational predictions about the conformational preferences of these amino acid residues in the unfolded state in water. Our results indicate these conformational preferences are the result of the balance between pPII-stabilizing backbone–water interactions and repulsive side chain–backbone interactions where the latter will depend uniquely on the atomic makeup and geometry of the side chain.

## Introduction

Classically, protein folding theory states the amino acid residue sequence determines the three-dimensional native structure, and the biological function, of the protein. However, the driving forces of the folding process, particularly in the early stages which lead to the molten globule state,^1^ remain elusive due to the high dimensionality of the phase space.^2^ The established main forces contributing to chain collapse and folding are the hydrophobic effect and intramolecular backbone interactions.^3^ The discovery of intrinsically disordered proteins (IDPs), which have no native fold and can still perform biological function, required an expansion of the classical idea of protein folding and dynamics. It has been shown the dynamics of IDPs are dependent on properties like hydrophobicity and net charge, which are properties determined by the protein sequence,^4^ and restricting conformations of amino acid residues in IDPs have been shown to have sequence dependent effects on the resultant conformational ensemble.^5^ While IDPs are typically characterized by low hydrophobicity and high charge density,^6^ Baxa et al. recently showed a protein, which has amino acid residue composition of a protein expected to collapse and fold, is actually an IDP.^7^ Their results indicate that hydrophobic forces are insufficient to alone describe initial chain collapse in protein folding^7^ and imply that sequence-dependent effects need to be more rigorously characterized to understand why proteins with similar amino acid composition can exhibit drastically different dynamics.

Understanding the fundamental mechanisms which govern local conformational preferences in the unfolded state within proteins can lead to a better understanding of protein folding for natively folded proteins, the lack of folding in IDPs, and to innovations in fields such as peptide and protein design. Ramachandran and Ramakrishnan demonstrated chiral amino acids can only sample a restricted region of Ramachandran space.^8,9^ The Flory random coil model posits that each amino acid residue in a chain would sample the available configurations in Ramachandran space independently of other residues in the chain.^10^ However, this random coil model is at odds with both μs-scale protein folding times^11,12^ and the unique dynamics exhibited by IDPs with different sequences.

The discovery of IDPs, and their sequence-dependent properties, led to many investigations of the conformational preferences of blocked dipeptides and short oligopeptides over the past 25 years to characterize and understand conformational preferences in the unfolded state. The reader is referred to a comprehensive review by Schweitzer-Stenner for more detail on this topic^13^ but some notable results are summarized here. Alanine, in particular, strongly prefers the local polyproline II (pPII) state in water. Repeat amino acid residues adopting a pPII conformation leads to a pPII helix which can be found in glycine-rich proteins such as the snow flea antifreeze protein and collagen.^14,15^ The local pPII state, which is the focus of this work, refers only to the conformation of an individual amino acid residue and is typically characterized by the region of Ramachandran space adjacent to the β states. Other amino acids exhibit amino acid residue-specific balances between pPII and β states.^16−20^ Toal et al. used a two-state model to describe the pPII-β balance of 15 guest amino acid residues in GxG peptides from NMR data at different temperatures and show amino acid residue-specific conformational preferences, rather than uniform sampling of available Ramachandran space. The authors observed an isoequilibrium between pPII and β states in all central amino acid residues which was argued to imply a common mechanism, peptide solvation, driving conformational preferences in all residues.^17^ A later study by Zhang et al. analyzed AcLxPNH_2_ peptides with NMR and determined there was a direct correlation between peptide solvation and pPII stabilization.^21^

Computational tools are crucial for understanding atomistic mechanisms governing these experimentally observed conformational preferences of amino acid residues and oligopeptides in the unfolded state. Ilawe et al. performed DFT calculations of central A, V, L, and I in GxG peptides, surrounded by ten explicit water molecules, and determined the pPII state was enthalpically stabilized and entropically destabilized, which lead to amino acid-specific propensities for β states.^22^ Meral et al. conducted Molecular Dynamics (MD) simulations, using the OPLS-AA force field, of various GxG peptides and determined the pPII state was associated with higher backbone hydration than β states.^23^ Zhang et al. investigated the dynamics of central alanine in GAG peptides using multiple MD force fields and found that the pPII state corresponded to the state with the most peptide–water hydrogen bonds.^24^ Yuan et al.^25^ used adaptive force matching to simulate a blocked alanine dipeptide in water. They found the β state leads to polarization frustration in the solvent while the pPII state leads to polarization stabilization and dipole cooperativity, and is ultimately the lower free energy state for alanine.

While these results have adequately explained some experimental observations, computational tools have proven to be quite limited in elucidating the atomistic mechanisms driving the amino acid-specific conformational preferences, such as those observed by Toal et al.^17^ Quantum mechanical methods are computationally expensive and can only tractably solve very small systems (≈100 atoms), limiting their utility to analyzing a small set of structures (not full trajectories) with maybe a few water molecules. Particularly troublesome are amino acid residues with bulky side chains because they have more atoms and more water molecules are required in the solvation layer to capture solvation effects.^22^ Hybrid QM/MM simulations of short oligopeptides may be tractable but still computationally expensive and are limited to small system sizes. Monte Carlo methods are limited by the combinatorics of the problem, even for single amino acid residues with longer side chains, and the choice of Hamiltonian can produce conformational preferences which are at odds with experimental observations.^26^ There are various machine learning methods for protein structure prediction, such as AlphaFold.^27^ However, the application of structure prediction tools like AlphaFold to IDPs or disordered protein regions must be approached with caution since they typically do not generate ensembles of conformations, as noted by Ruff and Pappu.^28^ There are machine learning tools trained to characterize IDPs specifically, such as SeqDYN,^29^ which have been shown to identify how different amino acid compositions in protein regions can affect observed experimental results. However, machine learning tools predict outcomes based on identified patterns in training sets and do not typically elucidate underlying physical mechanisms driving the resultant conformations or conformational ensemble.

MD appears to be the tool most applicable for characterizing the dynamics of amino acid residues in the unfolded state. However, like with Monte Carlo methods, the accuracy is limited by the underlying Hamiltonian. The aforementioned MD study by Meral et al. and a recent MD study by Andrews et al. showed multiple, commonly used additive MD force fields overly promote the pPII structure in many amino acid residues in short oligopeptides and do not accurately produce the expected amino acid-specificity in the Ramachandran distributions expected from experimental data.^23,30^ Another recent study by Andrews et al. showed that two polarizable force fields did not improve the reproduction of experimental data relative to classical, additive models for glycine and alanine.^31^ Jiang et al. showed via coil libraries that the backbone dihedral and side chain rotamer distributions are very closely related and that force fields poorly captured these relationships.^32^ While the work of Jiang and collaborators used older force fields, it has not been shown, to the best of our knowledge, that more modern force fields do adequately capture this relationship. There have been a variety of efforts to change force field parametrizations to fix the overpromotion of pPII and other issues^33−41^ with mixed or limited success in generally improving the force fields. The high number of parameters and the difficulties in incorporating experimental data in the parametrizations of force fields makes the task of identifying target parameters to adjust, while avoiding unwanted side effects, quite difficult. There are a number of reviews in the literature which summarize some of these issues and some potential ways forward.^33,42,43^ It is worth noting there are other force fields which make some unique changes to their Hamiltonian which may improve results, including coupling terms for backbone and side chain dihedral potentials, which should be more thoroughly tested for short oligopeptide systems.^36,37^ Without a clear path forward for improving the force fields, other means of investigating the atomistic mechanisms driving conformational preferences of amino acid residues in the unfolded state should be explored.

Another potential way to investigate these systems involves reducing the number of parameters, at least with respect to the large number present in MD and QM methods. The question is then how to achieve this. For protein structure determination and peptide conformational preferences, the backbone dihedral angles are of primary importance. For an individual amino acid residue without any nonlocal interactions, there are two main contributions: intrapeptide and peptide–water interactions. Both groups contain many degrees of freedom such as side chain torsions, vibrational degrees of freedom, orientation of hydration layer water molecules and bulk water effects. However, there are things we know about nonglycine and nonproline amino acid residue structures which are persistent such as chirality the tetrahedral structure of the backbone, and the planarity of the peptide bond. These factors could likely be excluded in a model attempting to explain purely backbone dynamics and alleviates the need to preserve these qualities. Additionally, the torsional degrees of freedom have been shown to account for 98% of the mean-square fluctuation of protein dynamics.^44^ A reasonable question is then to ask if a local intrapeptide mean field can be created by averaging over the remaining torsional degrees of freedom in the side chain for each backbone dihedral angle orientation and if that could explain the conformational preferences of the backbone for different amino acid residues in the unfolded state.

There is some data which indicates this could be possible. Zhou et al. extracted amino acid mimetics from crystal structures with β and α-helical backbone configurations, rotated the side chain torsion angles, and calculated rotamer distributions using states which did not include steric clashes (i.e., atomic overlaps). This procedure led to the accurate prediction of rotamer distributions for amino acid residues with nonpolar side chains in folded proteins.^45^ This work agrees with the results from Jiang and collaborators^32^ and shows more explicitly that local interactions contribute to the relationship between the backbone and side chain structural preferences. The work of Zhou and collaborators was done on amino acid residues in folded proteins, which means the backbone dihedral angles were stabilized by nonlocal intramolecular interactions and, therefore, acted as constraints on the side chain rotamer distributions. In the unfolded state, the backbone is no longer stabilized by enthalpic, nonlocal, intramolecular interactions. Therefore, short-range, repulsive interactions between the backbone and side chain should also have an effect on the backbone distribution of the residue, which can be unique based on the chemical and geometrical properties of the side chain. Otherwise, the system would suffer an entropic penalty, through side chain rotamer state restriction, with no enthalpic gain from the many nonlocal intrapeptide interactions involving the backbone in a folded protein environment.

The work presented here takes inspiration from the analysis of Zhou and collaborators^45^ by applying a similar analysis but also varying the backbone dihedral angles to probe which states in Ramachandran space which result in the fewest clashes between the side chain and backbone for alanine and the branch chain amino acids valine, leucine, and isoleucine. These amino acids were chosen because they lack net charge, are not polar (no enthalpic side chain-water or side chain-backbone interactions), and each side chain is composed solely of carbons and hydrogens. Despite their similarities, each amino acid residue considered has quite unique intrinsic conformational preferences and the reason for this has not been explicitly established. The work presented here will show that A, V, L, and I will have unique landscapes of atomic overlaps, averaged over side chain torsion configurations, in Ramachandran space, indicating unique intramolecular energy landscapes which could affect their conformational preferences.

To this end, the above analysis was then repeated, but the average intramolecular Lennard-Jones energies are calculated in Ramachandran space for each backbone dihedral orientation rather than number of atomic overlaps. Then, an empirical potential landscape representing the mean peptide backbone-water hydrogen bonding interactions is adapted from a Gaussian model Ramachandran for alanine in GAG which was developed by Schweitzer-Stenner,^16,24,46^ and added to the Lennard-Jones energy landscape in Ramachandran space. From this perspective, the pPII state can be considered as the “ground state” of the amino acid residue backbone in the unfolded state in water, enthalpically stabilized by backbone-water interactions, and is then perturbed by short-range repulsive forces between the backbone and side chain of the amino acid residue. These repulsive forces depend explicitly on the chemical and geometrical properties of the side chain. This is consistent with the concept of entropic destabilization of the pPII state presented by Ilawe et al.^22^ Our work will show that these combined effects are sufficient to achieve amino acid residue-specific conformational preferences without introducing dihedral-specific energy terms to the Hamiltonian. This analysis only characterizes the minima of the resultant landscape, which informs conformational preferences alone, and does not capture dynamics or characterize kinetic rates between states. Resultant Ramachandran distributions derived from the mean fields will be compared against previously reported NMR coupling constant values.^16,19^ Future work will involve exploring how sequence dependent effects can emerge from the intrinsic conformational preferences of individual amino acid residues.

## Methods

### Generating Amino Acid Mimetic Structures

PDB structures of tripeptides GAG, GVG, GLG, and GIG were constructed using the protein builder function of the molefacture feature in the Visual Molecular Dynamics (VMD) software package.^47^ CONECT records in the PDB were generated using trjconv function of the GROMACS software suite.^48−54^ Then, the PDB files were manually manipulated in three ways. First, the -C’ angles of the central residues were set to 110^◦^, using the molefacture tool of VMD, to be consistent with calculations done by Ramachandran and Ramakrishnan.^8^ All atoms of the first and third amino acid residue were deleted except for the hydroxyl group (C=O) of the N-terminal glycine and the amine group (N–H) of the C-terminal glycine, resulting in amino acid residue mimetic structures. The ends of the mimetic structure are considered electrostatically neutral. Atom numbers and CONECT records are renumbered so the first atom number, referring to the C’ of the first residue, is one. Relevant PDB files used for these analyses are available on Github.^55^

### Calculating Atomic Overlaps in Ramachandran Space

The PDB of each mimetic (A, V, L, and I) is used as input to the stericScan function of Dihedral Rotation of Proteins (DROP) software,^55^ introduced in this publication. Atoms were considered to be overlapping if the distances between atomic centers were less than those defined by Ramachandran and Ramakrishnan,^8,9^ henceforth denoted R_*s*_. Atomic overlaps were only considered for atoms which 4 or more covalent bonds apart. Then, for a given backbone conformation or ϕ/ψ angle pair, rotation operators were applied to the side chain torsional angles in a nested fashion. Starting with the outermost, relative to the backbone, side chain torsional angle (i.e., χ_2_ for isoleucine), the torsional angle was rotated 360^◦^ in 2^◦^ increments. After every incremental rotation of the outermost side chain torsional angle, the number of atomic overlaps are calculated and added to an aggregated total. After the outermost side chain dihedral is rotated by 360^◦^, the next side chain torsional angle is rotated 2^◦^ (i.e., χ_1_ for isoleucine) and the rotations of the outermost χ angle is repeated. This process continues until the χ_1_ angle is rotated 360^◦^ in 2^◦^ increments. The total number of atomic overlaps counted for all side chain configurations is then normalized by a factor of  where *N*_χ_ is the number of side chain torsional angles present in the amino acid residue. This procedure is repeated for all of backbone, or Ramachandran, space in 2 × 2^◦^ bins creating a Ramachandran distribution of average number of atomic overlaps in an amino acid for a given backbone configuration. Only standard torsion angles were considered so the orientations of methyl (CH_3_) groups were not changed.

### Generating the Mean Fields

#### Intramolecular Lennard-Jones Interactions

The ϵ and σ values for the Lennard-Jones potential are taken from the CHARMM36m force field.^56^ Atom types are not used so, for example, each carbon in the mimetic have the same LJ parameters. The values used for different atom pairs are determined using the Lorentz–Berthelot combination rules.^57^ The average Lennard-Jones potential values are calculated via the same procedure as the atomic overlaps, averaging energies instead of number of atomic overlaps. Structures with large or many atomic overlaps lead to large energies and energy gradients, resulting in deep, narrow free energy minima. For example, N–N interactions at 1.5  and 1  result in energies of 42 000 kJ/mol and 5.4 × 10^6^ kJ/mol, respectively. Structures including these interactions would not be expected to be observed in experiments. Defining a cutoff for excluding such structures is not as clear as defining a hard-sphere overlap since the LJ potential is continuous and positive potential values are expected but the maximum value allowed is unknown. Therefore, structures are only considered if the distances between each backbone-side chain atom pair, which are at least four covalent bonds apart, are greater than λ*R*_*s*_ where λ is treated as a hyperparameter and R_*s*_ is the cutoff for an atomic overlap for the atom pair in question as defined in the previous section. Only distances between backbone and side chain atoms are considered so that the high energy regions of Ramachandran space are preserved (i.e., the bottom right quadrant of Ramachandran space). Higher λ values result in fewer structures considered in the averaging and λ = 0 means no structures are excluded from averaging. If any point in Ramachandran space results in no valid side chain configurations, which means there is always a distance between a backbone and side chain atom less than γ*R*_*s*_, this configuration is given an energy of 500,000 kJ/mol. This is an arbitrarily chosen value large enough to preserve higher energy regions of Ramachandran space. To explore the effects of some vibrational degrees of freedom on the calculations, mean fields were also calculated where the N–C_α_−C’ angles were set to 105° and 115° degrees.

#### Peptide–Water Interactions

To account for peptide–water interactions, an empirical Gaussian model Ramachandran distribution of alanine, as constructed by Schweitzer-Stenner,^16,24,46^ is used. The Gaussian model Ramachandran distribution is generated by producing a linear combination of Gaussian subdistributions in Ramachandran space where each subdistribution is identified by the peak center in Ramachandran space and widths. The location and number of peaks are chosen to best fit a comprehensive set of experimental data. The parameters for producing the Gaussian model Ramachandran distribution of central alanine in GAG are found in Hagarman et al.^16^ The Ramachandran distribution is converted to a free energy landscape in Ramachandran space using Boltzmann factors
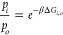
1where p_*o*_ is the reference probability which is the lowest value in the Ramachandran distribution so that the least populated regions will have a free energy of zero and the most populated basin will have a negative free energy. T will be set to 298 K and β will then be 1/2.479 (kJ/mol)^−1^.

#### Ramachandran Distributions from the Mean Field

The average LJ energies and the peptide–water energies will be summed to generate the combined mean field for each amino acid residue mimetic. eq 1 will be used to convert the combined mean energy landscape back to a Ramachandran distribution, or a normalized probability distribution in two-dimensional space where the two independent variables are the backbone dihedral angles, where p_*o*_ will be used as a normalization factor. The resultant Ramachandran distributions are useful for determining properties, such as basin locations, depths, and widths, of the constructed energy landscape. It should be noted that these resultant Ramachandran distributions are not derived from any dynamical data.

### Analysis of Ramachandran Distributions

#### Definition of Backbone Mesostates and Mesostate Populations

The following mesostate definitions are used to be consistent with prior works:^24,30,58^ (a) pPII (, ), (b) antiparallel β-strand or aβ (, ), (c) the transition region between aβ and pPII or βt (, ), parallel β-strand or pβ (, ) (e) right-handed α-helix (, ). Mesostate populations are then calculated by integrating the Ramachandran distribution probabilities within the defined region of Ramachandran space corresponding to the mesostate.

#### Calculation of NMR Coupling Constants with Karplus Equations

Ramachandran distributions can be used to calculate NMR J-coupling constants using the empirical Karplus relations which is defined as
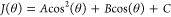
2where θ can correspond to ϕ or ψ depending on the J-coupling constant being considered. To calculate the experimental observable J-coupling constant, an ensemble average has to be computed over Ramachandran space.
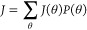
3

P(θ) refers to the one-dimensional distribution in Ramachandran space for the angle ( or ψ) depending on which J-coupling constant is of interest. Here, two J-coupling constants are calculated, ^3^ and^1^, which depend on the ϕ and ψ backbone angles, respectively. The Karplus parameters used to calculated ^3^ and ^1^ are from Wang and Bax^59^ and Wirmer and Schwalbe,^60^ respectively. These calculated values are then compared with previously reported experimental and MD-derived results.^16,30^

## Results

**Figure 1:**
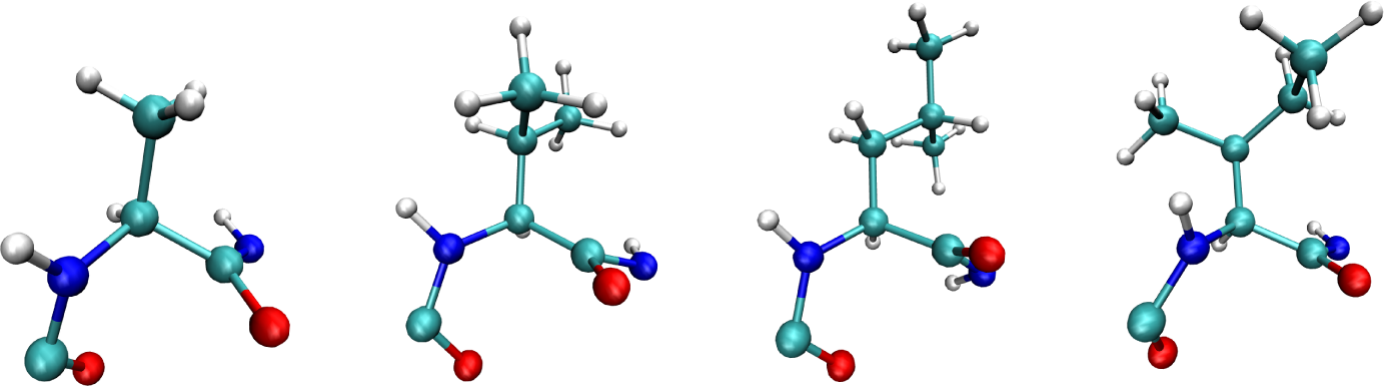
Visualizations of the amino acid residue mimetic structures of (from left to right) alanine, valine, leucine, and isoleucine. Visualizations were made with VMD.^47^.

In this work we explore the effects of average peptide–water and intrapeptide LJ interactions on the backbone conformational preferences of amino acid residues alanine, valine, leucine, and isoleucine in unfolded polypeptide chains. To simulate an amino acid residue in an unfolded polypeptide chain, as opposed to a blocked dipeptide or equivalent, mimetic structures were used which include the C and O atoms from a preceding amino acid residue and the N and H atoms from a proceeding amino acid residue. The inclusion of the extra atoms from adjacent residues are important to consider because they should contribute to the sterics for central residues with longer side chains. All calculations are then performed assuming no nonlocal, intrapeptide interactions are present. The mimetic structures are visualized in Figure 1. Two analyses are conducted and both of which involve averaging quantities over all side chain configurations for each backbone dihedral configuration. The first is calculating the average number of atomic overlaps, or steric clashes, in each backbone configuration. The second analysis calculates the average intrapeptide LJ energy for each backbone configuration and adds an empirical peptide–water energy term to create a mean field from which Ramachandran distributions can be derived and compared to previous results. These results will show is that unique conformational preferences of amino acid residues are the result of competition between peptide backbone-water and repulsive intrapeptide interactions (see Methods for more details).

### Atomic Overlaps in Ramachandran Space Are Unique to Each Amino Acid Residue Mimetic

**Figure 2:**
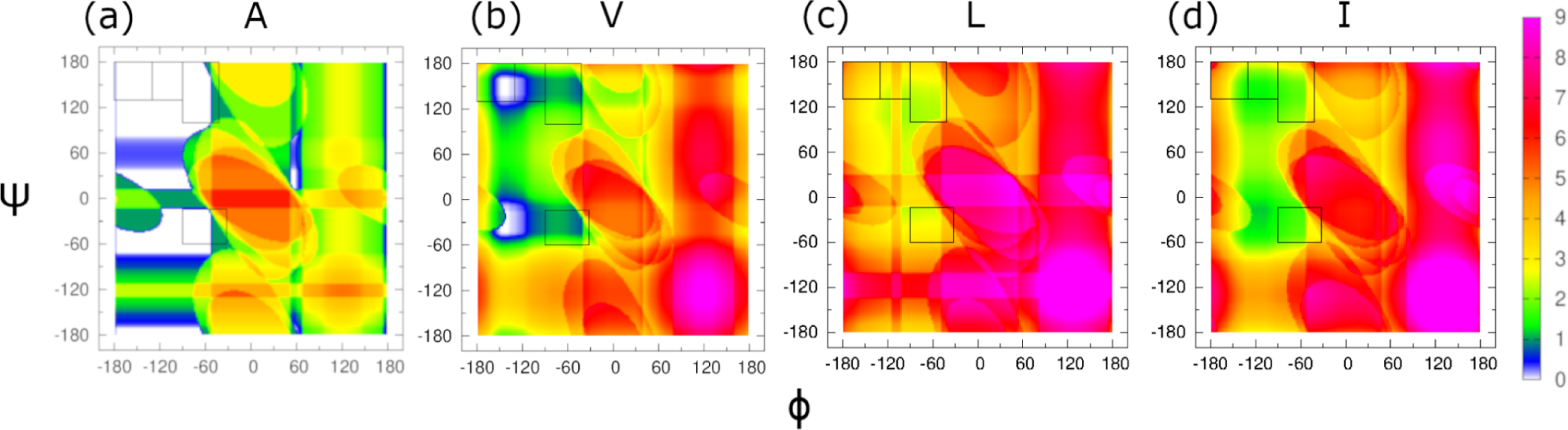
(a–d) Average atomic overlaps in Ramachandran space for alanine, valine, leucine, and isoleucine amino acid mimetics.

Figure 2 shows the average number of intrapeptide atomic overlaps in Ramachandran space for peptide mimetics alanine, valine, leucine, and isoleucine. Table S1, reproduced from Ramachandran,^8^ shows the radii considered in the calculations. The *z* axis indicates the number of atomic overlaps normalized by the number of side chain configurations sampled for each pair of backbone dihedral angles. The boxed regions differentiate proximate mesostate regions, as described in Methods, and are shown with labels in Figure S1. The white areas in each plot in Figure 2 indicate regions of Ramachandran space with no atomic overlaps for any side chain configuration sampled.

Figure 2a shows the calculated atomic overlaps for alanine, as defined in Methods, and regions with few or no atomic overlaps are consistent with those reported by Ramachandran and Ramakrishnan^8^ as well as Finkelstein and Ptitsyn.^61^ There are a small number of atomic overlaps observed around ψ = 60 in Figure 2a which are not observed in prior works. Additionally, the size of some accessible regions in each Figure 2a differ in size relative to previous results such as the left-handed helical region () and to the left of the right-handed helical region (). While these are notable differences, both calculations presented here and in prior works predict intrapeptide steric clashes will not occur in similar regions of Ramachandran space for alanine.

Figure 2b–d shows the distribution of average atomic overlaps in Ramachandran space for amino acid mimetics valine, leucine, and isoleucine, respectively, as defined in Methods. There are similarities between Figure 2a–d in the proximate regions (i)  for all ψ and (ii)  where , which represent regions with high energies due to backbone atoms being in close proximity. For each amino acid residue mimetic, region (i) consists of peaks similar shape but different magnitudes. The steric clashes captured in alanine are present in the residue mimetics with larger side chains and the larger side chains contribute more steric clashes when averaged over all side chain configurations. The rest of the discussion in this section will focus on the region of Ramachandran space defined by  and , which contain the relevant regions for standard secondary structures.

The results for valine (Figure 2b) show that there are atomic overlaps in certain structures when the backbone is in the pPII state and no atomic overlaps when the backbone is in some aβ and βt configurations. This indicates that β states minimize atomic overlaps and, therefore, intrapeptide energy. In contrast to alanine (Figure 2a), there are no atomic overlaps in the region near ϕ = −130, ψ = −30. This is likely is the result of the longer bond length between the  and  atoms in valine than the  and  atoms in alanine, which means the backbone and side chain atoms are farther apart in this backbone configuration for valine. The landscape of average intrapeptide atomic overlaps in Ramachandran space for the leucine mimetic is quite different than that of valine (Figure 2c). No region of Ramachandran space is without atomic overlaps. The regions with the fewest average atomic overlaps reside in the lower pPII regions and the right-handed helical regions. Additionally, there is a small region at  where the number of average atomic overlaps is greater than the surroundings indicating a potential barrier between the pPII and β backbone configurations. As for valine and leucine, the landscape of steric overlaps in Ramachandran space is unique for isoleucine as well (Figure 2d). Similar to leucine, there is no region in Ramachandran space free of steric clashes. However, there are fewer steric clashes on average in the pPII, β, around the α-helical region, and even in the γ turn region between pPII and α relative to leucine. In contrast to valine, isoleucine has many more intrapeptide steric clashes if the backbone is in an aβ conformation, and generally when .

Importantly, the distributions of average number of intrapeptide steric clashes in Ramachandran space, averaged over all side chain torsional configurations, are unique for each of the four amino acid residue mimetics. The landscape of steric clashes were additionally normalized by the number of atoms in the mimetic structure to determine if the different number of atoms in each mimetic are effecting the resultant steric landscapes and the results are shown in Figure S2. The overall shape of the steric landscapes are the same as in Figure 2 although the scale of the *z* axis has changed which indicates the number of atoms in these mimetics are not the origin of the unique steric landscapes. Therefore, the intrapeptide Lennard-Jones energy landscape in Ramachandran space will also be unique for each amino acid residue mimetic. These unique intrapeptide interactions could, in conjunction with peptide–water interactions, lead to observed intrinsic conformational preferences for amino acid residues in the unfolded state in water as observed experimentally.^17,30^

### Amino Acid Residue-Specific Ramachandran Distributions from Lennard-Jones and Peptide–Water Mean Field Interactions

We then generate energy landscapes in Ramachandran space using two contributing factors: intrapeptide LJ potential averaged over the side chain degrees of freedom for each backbone configuration and an empirical potential to represent the average of peptide backbone-water interactions. The Lennard-Jones parameters used in the calculations are in Table S2. To avoid large atomic overlaps, leading to large energies and energy gradients in Ramachandran space, a cutoff was implemented between side chain and backbone atoms that were at least four covalent bonds apart. A hyperparameter, λ, is used so that the cutoff is tunable and its effect on the energy landscape could be explored. Figure S3 shows an example of the contributions to the energy landscape from the average LJ energy calculations, the peptide–water energy landscape, and their sum for λ = 1. Other raw data is available in the github repository with scripts for plotting.^55^ Multiple mean fields were generated using values of λ in the range [0.70, 1.00] in intervals of 0.05. Mean fields were also generated for λ = 0.

**Figure 3:**
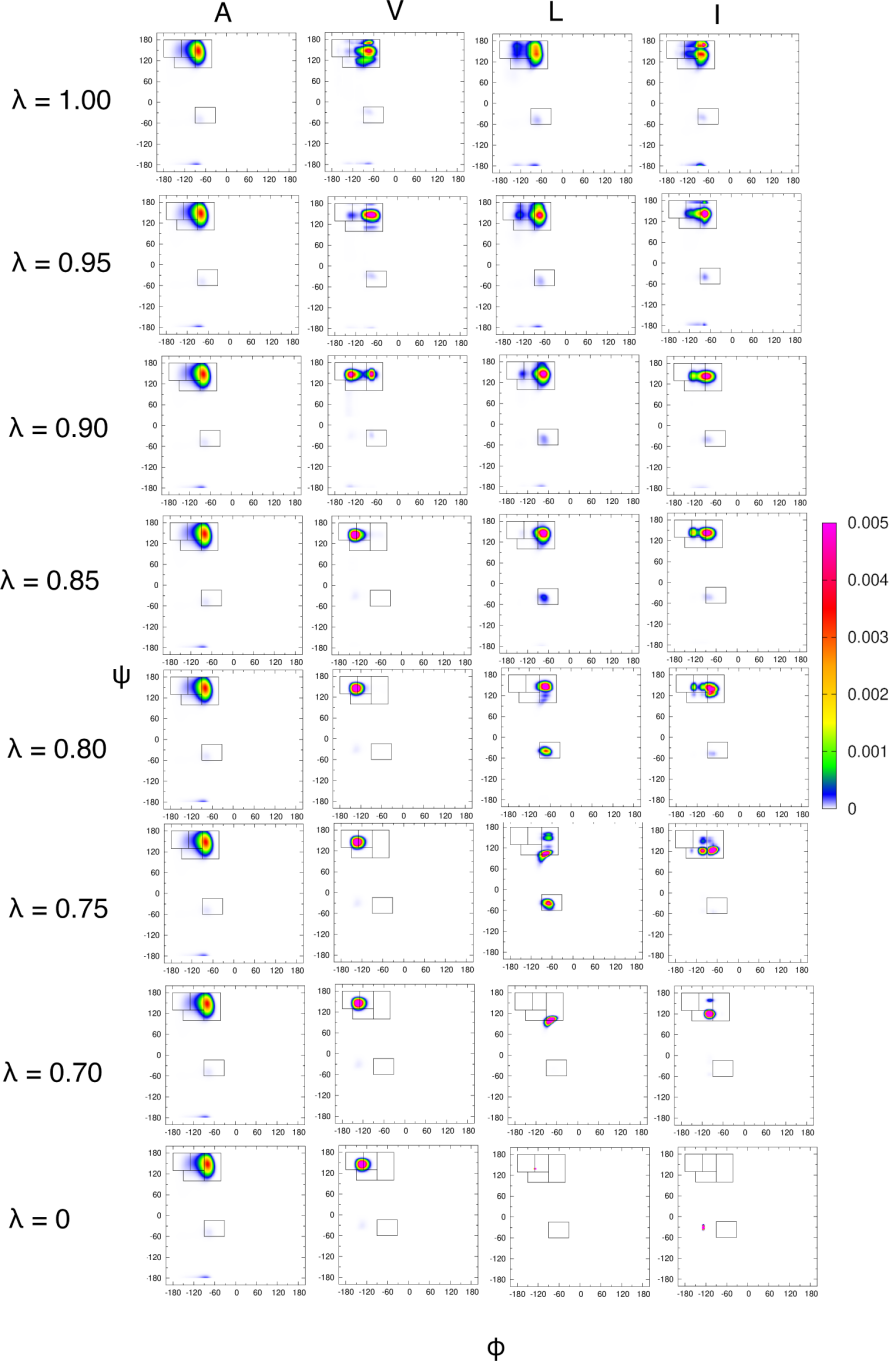
Ramachandran distributions, derived as defined in Methods, for amino acid residue mimetics of alanine, valine, leucine, and isoleucine for multiple different values of λ. λ = 0 means all structures were considered regardless of atomic overlap. Note that these Ramachandran distributions are calculated from a mean field energy landscape using Boltzmann factors and represent the energy landscape minima but include no dynamical data.

To elucidate properties of the minima of the resultant energy landscapes in Ramachandran space, each energy landscape was converted to a Ramachandran distribution using Boltzmann factors as described in Methods. Note that these Ramachandran distributions are not derived from dynamical data and are only used to identify and compare minima in the corresponding energy landscapes. The resultant Ramachandran distributions for each amino acid residue mimetic and each value of λ are shown in Figure 3.

The Ramachandran distribution for alanine (Figure 3, first column) shows very little dependence on the value of λ which is unsurprising because the minimum of the energy landscape is in a region of Ramachandran space where Figure 2a show there are no steric clashes. If no steric clashes are observed, changing the value of λ then has no effect on the results of the calculation for a given backbone dihedral angle pair. This is not the case for any of the other amino acid residue mimetics analyzed in this work.

For valine (Figure 3, second column), higher values of λ result in pPII-dominated Ramachandran distributions, similar to that of alanine. However, as λ decreases, the Ramachandran distribution becomes narrower in the ψ dimension and transitions from primarily pPII, to a pPII-β balance, to purely β mesostates. The location of the energy minimum, or maximum of the Ramachandran distribution, does not appear to shift in ϕ or ψ as a function of λ. The inverse relationship between λ and the β mesostate population indicates that the side chain conformations which result in a higher LJ energies perturbs the enthalpically stabilized pPII state in favor of β states. The Ramachandran distributions do not significantly change for values of λ ≤ 0.85.

The Ramachandran distributions for leucine (Figure 3, third column), much like the results of Figure 2c and S3 would suggest, contrast from the Ramachandran distributions of the other amino acid residue mimetics. For higher λ, there are similarities in that the Ramachandran distribution is pPII-dominated. However, there is a nontrivial β population. As λ decreases, the distribution becomes narrower along the ψ axis and no substantial shift in the maximum of the Ramachandran distribution is observed, as was the case with valine. In contrast with valine, the β population decreases with λ in favor of α helical populations and is the only amino acid residue considered in this work to have a substantial peak in the α region. It has been shown from coil library studies that leucine is found more frequently in helical structures compared to the other branch chain amino acids.^62,63^ For λ values less than 0.80, a third peak appears in the lower pPII region bordering on the γ turn region. This third peak aligns with the area associated with the minimum average number of atomic overlaps for leucine in Figure 2. At λ = 0.70, the pPII peak higher in ψ and the α peak are absorbed into the new peak at the bottom of the pPII region. Of valine, leucine, and isoleucine, leucine is the only amino acid residue whose Ramachandran distribution does not reduce to a β mesostate for low λ values. When λ is set to zero, the distribution becomes overly constrained to a very narrow peak in the βt region of Ramachandran space.

The Ramachandran distributions for isoleucine (Figure 3, fourth column) also deviate from each of the other amino acid residue mimetics. For high λ, the Ramachandran distribution is dominated by pPII mesostates. As λ decreases, the main peak narrows along the ψ axis, as observed for valine and leucine, but a substantial shift in the -ψ direction is observed. Like valine, lower λ values yield a transition from pPII to β mesostates but the location of the β peak appears in the pβ region of Ramachandran space which is consistent with experimental data.^30^ When λ is set to zero, the distribution becomes overly constrained to a very narrow peak to the left (more negative in ϕ) of the α helical region. For both leucine and isoleucine, very low λ values (i.e., λ ≈ 0) result in drastic changes to the Ramachandran distribution because the longer side chains allow for configurations where backbone and side chain atoms can heavily overlap (see Figure S5 for visual examples) and should not be considered in the construction of the energy landscape.

**Figure 4:**
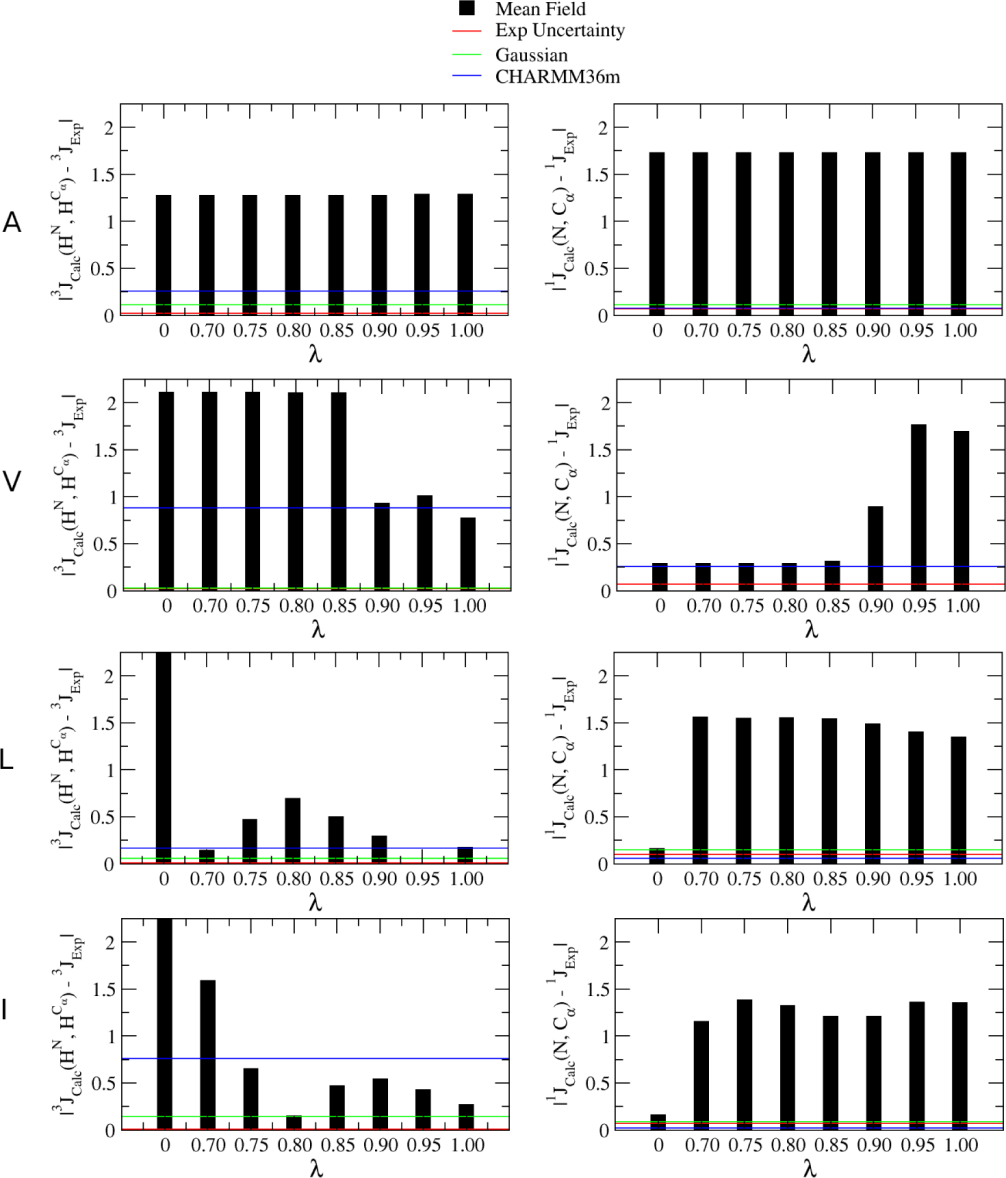
Absolute value of differences between calculated and experimental ^3^ and ^1^ for different values of λ. References values for experimental uncertainty (red), values calculated with the Gaussian Ramachandran (green), and values calculated using Ramachandran distributions generated with the CHARMM36m force field (blue) are shown for reference.

To explore the effects of λ more systematically, the Ramachandran distributions in Figure 3 were used to calculate NMR coupling constants and were then compared to the corresponding experimental values. The results of this calculation are shown in Figure 4. Red, green, and blue lines in each figure refer to the experimental uncertainty, the values calculated from the Gaussian model Ramachandran, and the values calculated from CHARMM36m-derived Ramachandran distributions, respectively. For alanine, the value of λ does not strongly affect the resulting coupling constants. In the case of valine, lower λ values result in higher deviations from the experimental ^3^ due to loss of pPII and gain in β populations but also leads to a shift in ψ which reduces the error associated with the ^1^ value. The interpretation of these results for leucine and isoleucine are less clear. In both cases, with exceptions for λ = 0, the calculated ^1^ does not change significantly for different λ values. Conversely, the calculated ^3^ values for leucine and isoleucine are quite sensitive to the value of λ. Calculated ^3^ values are close to experimental values for multiple values of λ, 0.70 and 0.95 for leucine and 0.80 and 1.00 for isoleucine, and values in between produce coupling constants which deviate further from experimental values. As ^1^ exhibit low sensitivity to λ values, it is difficult to interpret which λ values are most appropriate for these two amino acid residues and comparisons with more experimental data would be required. However, since the calculated coupling constants from the Ramachandran distribution for alanine deviate significantly from experimental values, additional model refinement will be done before comparing to additional experimental measures.

The analysis to this point has omitted the vibrational degrees of freedom. To explore their effect on the resultant energy landscapes and Ramachandran distributions, additional Ramachandran distributions were generated with N–C_α_–C’ backbone angles of 105^◦^ and 115^◦^. The results of these calculations are shown in Figure S4. This angle was chosen for this analysis because its variation changes the distances between the peptide groups and the side chain, which will affect interaction strengths. As was the case for varying λ, the Ramachandran distribution for alanine only exhibits minor changes with backbone geometry. For the other three amino acid residues, more severe changes are observed. As the backbone angle increases, the free energy minimum is positively shifted in ψ and the distributions are generally more constrained to higher ϕ values. The energy minimum still resides in the pPII region of the Ramachandran distribution although these changes do result in a decrease of β populations. This analysis shows that including multiple geometries will have an impact of the energy landscape and resultant Ramachandran distributions and should be included in future iterations.

This analysis shows that each amino acid residue has a unique free energy landscape in Ramachandran space, leading to amino acid-specific conformational preferences qualitatively consistent with experimental predictions.^17,30^ The only interactions considered are attractive backbone-water and LJ intrapeptide interactions where the latter would be unique based on the chemical makeup and geometry of the side chain since the backbone is the same in each mimetic. Therefore, this work provides evidence the intrinsic conformational preferences of these amino acid residues can be directly attributed to the competing attractive backbone-water and repulsive backbone-side chain interactions.

### Ramachandran Distributions Share Qualitative Properties with Others Derived from Empirical and Molecular Dynamics Methods

**Figure 5:**
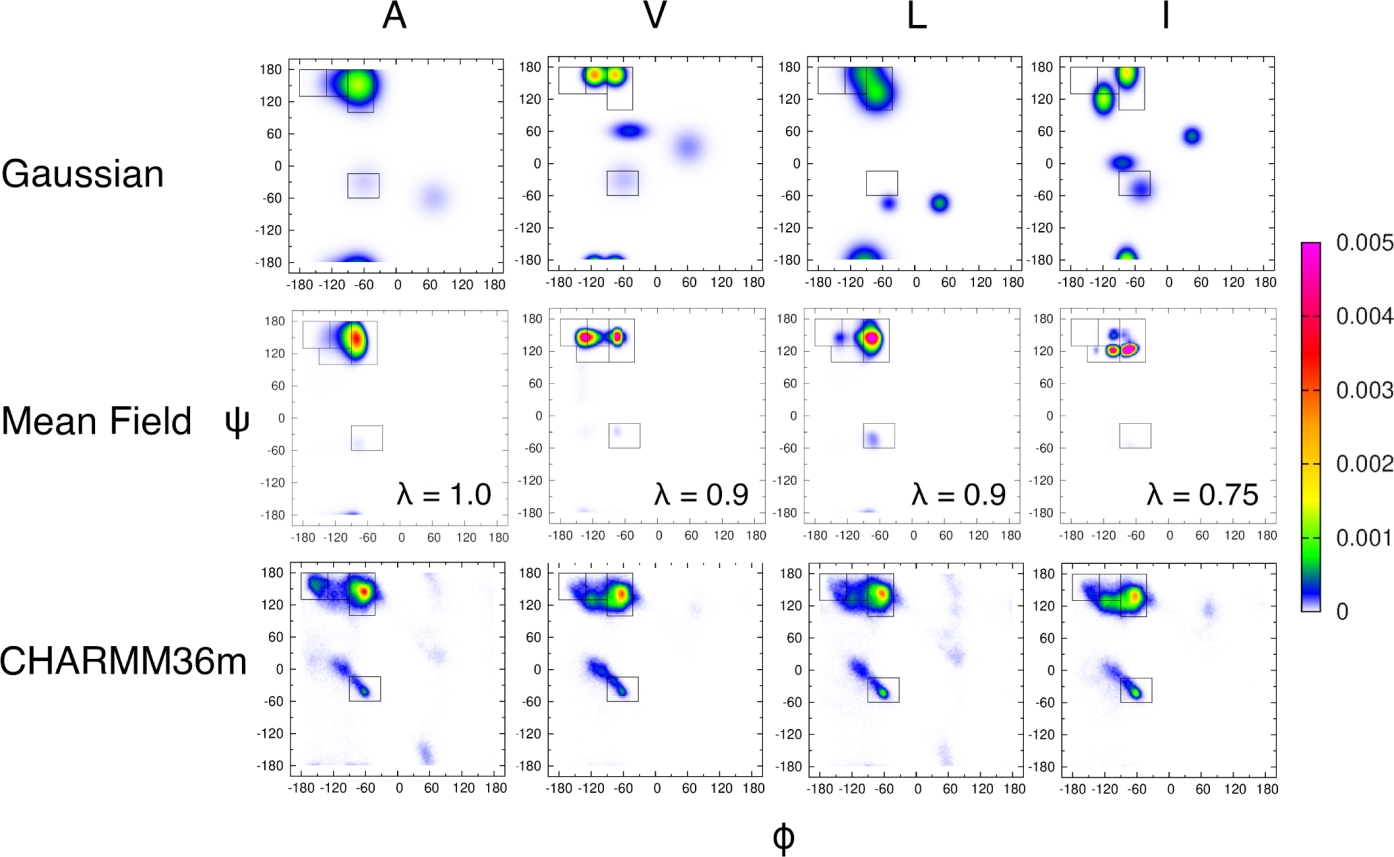
Ramachandran distributions for amino acid residues, from left to right, alanine, valine, leucine, and isoleucine derived from (top row) an empirical Gaussian model method, (center row) this work for specific values of λ, and (bottom row) the CHARMM36m molecular dynamics force field. The distributions derived from this work are in the center row to facilitate comparisons with the other two methods. Ramachandran distributions in the top and bottom rows are reproduced from ref. (30) with permission from the PCCP Owner Societies. Copyright 2022.

To assess the Ramachandran distributions derived in this work direct qualitative comparison to Ramachandran distributions derived from other methods and a direct quantitative comparison to experimental data was conducted. Ramachandran distributions for specific λ values where the amino acid residue exhibits qualitative properties consistent with experimental data are chosen: 1.0 for alanine, 0.90 for valine and leucine, and 0.75 for isoleucine. These four Ramachandran distributions are compared with distributions derived from the Gaussian model approach of Schweitzer-Stenner^46^ and molecular dynamics with the CHARMM36m force field from prior work^30^ in Figure 5. The Gaussian model and CHARMM36m derived Ramachandran distributions were compared in a prior work and will not be directly compared here.^30^

Generally, the Ramachandran distributions derived in this work (Figure 5, center row) have much narrower peaks compared to those derived from the Gaussian model and CHARMM36m. Aside from the multiple conversion steps used to generate the final Ramachandran distributions, the model in this work does not capture diffusive motion of the backbone degrees of freedom. CHARMM36m captures this explicitly and the Gaussian model implicitly by tuning the parameters of the distributions to experimental data. However, the locations of the Ramachandran distribution maxima can be directly compared through the prevalence of mesostates (Table S3) and NMR coupling constants (Tables S4 and S5). Visualizations of the Karplus relations with the parameters in Table S4 are shown in a study by Milorey et al.^64^

The Ramachandran distribution for alanine derived in this work in many ways directly reflects the Gaussian model Ramachandran distribution. Of course, the Gaussian model Ramachandran distribution was directly used as a component of the mean field. But, the lack of repulsive LJ energies in the pPII and β regions of Ramachandran space preserved the dominance of the pPII mesostate present in the Gaussian model. The Mean Field-derived Ramachandran distribution overestimates the pPII and βt populations (0.69, 0.24) and underestimates the α helical populations (<0.01). It does produce low aβ and pβ populations, consistent with the Gaussian model and in contrast with CHARMM36m. The high pPII and βt populations, the location of the pPII basin maximum, and the low α populations lead to a under and overestimation of ^3^ and ^1^, respectively.

In the case of valine, the mean field-derived Ramachandran distribution is also qualitatively similar to the Gaussian model Ramachandran. A prominent peak resides in each the pPII and β regions. However, there are stark differences. The peaks of mean field-derived Ramachandran in the pPII-β regions reside lower along the ψ axis than in the Gaussian model. However, this is consistent with the CHARMM36m-derived Ramachandran distribution. For λ = 0.90, the pPII population of the mean field Ramachandran (0.34) is fairly consistent with that of the Gaussian model (0.30). The peak in the β region is spread between the βt and aβ regions a total population of 0.64. The Gaussian model distribution shows a β peak which predominantly resides in the βt region and has a total β population of 0.42. Like in the case of alanine, the mean field underestimates α populations of alanine relative to the Gaussian model. The large β population, particularly aβ populations, results in an even larger ^3^ value than observed for alanine. An increase in this value relative to that of alanine is consistent with experimental results. But, the value from the mean field Ramachandran is still much higher than the reported experimental value. Similar to alanine, the low α populations results in a low ^1^ value. The shift of the pPII and β populations to lower ψ values, relative to the Gaussian distribution, likely also contributes to the low ^1^ value. For example, CHARMM36m overestimates the α population relative to Gaussian model but the CHARMM36m-derived ^1^ value is still lower than experimental values, indicating a shift in the pPII peak center can drastically shift the value of the coupling constant.

The mean field-derived Ramachandran distribution for leucine, as observed with the Gaussian model, deviates from the valine and isoleucine in that the β populations are much smaller and the populations are mostly contained in the pPII region of Ramachandran space. However, the narrow pPII distribution produced by the mean field leads to a severe overestimation of pPII populations for leucine (0.85) compared to both the Gaussian model (0.43) and CHARMM36m (0.51). The β populations are underestimated compared to both the Gaussian model and CHARMM36m, and the α population (0.02) is greater than derived from the Gaussian model (0.00) but less than derived from CHARMM36m (0.07). Because the mean field Ramachandran distribution is largely constrained to the pPII basin, the calculated ^3^ value (6.48) is much closer to the experimental value (6.78) than for other amino acid residues. However, the calculated ^1^ value is low in comparison with both the experimental value and those calculated from the Gaussian model and CHARMM36m.

The mean field-derived Ramachandran distribution for isoleucine includes two distinct peaks in the pPII and *beta*, particularly the pβ mesostate, region which is consistent with the Gaussian model. However, the peak associated with the pPII mesostate is shifted downward in ψ relative to the Ramachandran distribution produced by the Gaussian model, as was the case for valine, and the pPII population is severely larger than calculated from the Gaussian- and CHARMM36m-derived Ramachandran distributions. Interestingly, the mean field-derived Ramachandran distribution closely resembles that produced by Amber ff19SB due to the location of the two peaks and the underestimation of the α populations relative to the Gaussian model.^30^ An additional peak in the βt region, although with a small population (0.03), is observed in the mean field-derived Ramachandran which is not observed in the Gaussian model or any MD-derived distribution of isoleucine.^30^ The calculated ^3^ value for isoleucine using the mean field-derived Ramachandran distribution is much lower (6.82) than the experimental value or the value calculated from the Gaussian model. However, it is higher than the value calculated from CHARMM36m (6.71), which may be attributable to CHARMM36m’s underestimation of the pβ population. As in the case of the other amino acid residues, the calculated ^1^ value is much lower than the experimental value or those calculated from the Ramachandran distributions of the Gaussian model and CHARMM36m.

The Ramachandran distributions of the four amino acid residue mimetics derived in this work demonstrate that amino acid residue-specific Ramachandran distributions can be produced by using a simple mean field that averages over the intrapeptide and peptide backbone-water interactions which are qualitatively consistent to those derived from experimental results.^16,30^ Future refinements to the method should improve the ability to reproduce experimental observables.

## Discussion

The purpose of this work was to explore the origins conformational preferences of amino acid residues alanine, valine, leucine, and isoleucine in the unfolded state in water. A primary goal of this work was to explore the origins of amino acid-specificity in Ramachandran space with as few parameters and contributing interactions as possible while also avoiding unphysical terms such as dihedral potentials which are common in molecular dynamics force fields. To this end, landscapes of the average number of steric clashes, averaged over all side chain configurations, were calculated for each backbone configuration in Ramachandran space. Then, a mean field was generated by calculating the average LJ intrapeptide energies in the same way, and adding the resultant landscape to an empirical energy term for peptide–water interactions derived from a Gaussian model Ramachandran for alanine in a GAG peptide.^16,46^ In both cases, the results were unique to each amino acid residue, indicating that the competing enthalpic peptide backbone-water interactions and repulsive side chain-backbone interactions, the latter of which are based on the unique chemical makeup and geometry of the side chain, result in amino acid residue-specific conformational preferences in the unfolded state in water. While the current model poorly reproduces experimental NMR coupling constants, we were successful in using this model to generate Ramachandran distributions which qualitatively reflected expectations from experimental results.,^16,19,30^ For example, the unique locations of the β basins in Ramachandran distributions for valine (βt and aβ) and isoleucine (pβ) as well as the lack of a prominent β peak in the Ramachandran distributions for leucine are both captured in the presented model. This work presents evidence that the unique intrinsic conformational preferences of these amino acid residues in the unfolded state is driven by competition between enthalpic peptide backbone-water and repulsive backbone-side chain interactions.

As the model presented in this work is quite simple, there are many limitations of the presented method which are potential avenues for future development. Incorporating atom types could increase the accuracy of the resultant mean field in Ramachandran space. Notably, terms accounting for electrostatics and partial charges are omitted entirely from the model. Due to the systems considered, which are electrostatically neutral, and the various potential methods for estimating electron densities, electrostatics were not considered for simplicity. However, the future inclusion will be necessary for specific side chains but may also provide improvements to the results for the systems in this work.

Only a single structure of each amino acid residue is used to generate the mean field. Therefore, vibrational degrees of freedom such as varying bond lengths and bond angles, which can affect the short-range repulsive and attractive energies, and the mean field by extension, were omitted in this work. While the torsional degrees of freedom have been shown to account for 98% of the mean-square fluctuation of protein dynamics,^44^ additional calculations were performed to assess how the vibrational degrees of freedom would affect the results and conclusions of this work. These results showed vibrational degrees of freedom will likely substantially contribute to the conformational preferences of amino acid residues in the unfolded state. Therefore, a mean field averaged over the torsional degrees of freedom serves here as an initial investigation into the contributing factors of intrinsic conformational preferences in amino acid residues in the unfolded state and the potentials would be refined in future work by averaging over various molecular geometries. Some torsional degrees of freedom were also omitted, such as the rotation of methyl groups in the side chains to reduce the number of configurations considered, which could be included to further refine the mean field potential. Parameters for specific atom types were not considered to reduce the number of parameters.

The low value of λ required for the pβ mesostate to appear for isoleucine (0.75), compared to the value used for valine and leucine (0.90), needs to be resolved for this method to apply to all amino acid residues equally. For valine, λ values lower than 0.90 resulted in the disappearance of the pPII population which is not consistent with experimental results. Which configurations contribute primarily to the unique properties of the Ramachandran distributions for each amino acid residue may need to be more explicitly characterized. One potential path to investigate this is to use side chain rotamer libraries to weight specific torsional angles and their influence on the backbone when generating the mean field.

The method by which the effect of backbone-water interactions is included does not explicitly include the degrees of freedom and the potential changes of the dynamics in the solvation layer for longer side chains. This work intentionally focuses on side chains which will not strongly enthalpically interact with water or the backbone. Similar to observations in the works by Zhou and collaborators,^45^ this method is not expected to work for such systems and modifications will have to be added to the current model to capture the side chain-water and side chain-backbone interactions, as well as intrapeptide electrostatics. The peaks in the Ramachandran distributions in this work are too narrow and lack the qualities of diffusive motion expected of the system. An additional term to account for this must be added to better model the whole system and potentially could improve reproduction of experimental data.

The method proposed here currently only applies to individual amino acid residues and does not alone alleviate problems associated with the many degrees of freedoms in longer peptides and proteins. A mean field constructed by considering even just the dihedral or torsional degrees of freedom in longer peptides will quickly become intractable for larger systems and it would necessitate a mean field for each sequence combination. To alleviate some of the combinatorial burden, future development could introduce a coupling term between mean fields of individual amino acid residues to explore nearest neighbor interactions and long-range sequence-dependent effects in oligopeptides as potentially emergent phenomenon as a result of the coupling. However, the current lack of terms designed to sufficiently capture long-range intramolecular and other solvent effects may limit the model’s current effectiveness as the system size increases. This model and future work have the potential to help understand the underlying physical mechanisms driving dynamics of unfolded proteins, both in the context of IDPS and early stage protein folding.

## Supporting Information Available

Cutoff distances used for determining atomic overlaps (Table S1); Lennard-Jones parameters (Table S2); mesostate populations (Table S3); Karplus parameters (Table S4); NMR coupling constants (Table S5); mesostate labels in Ramachandran plots (Figure S1); average atomic overlaps or steric clashes normalized by the number of atoms in mimetic (Figure S2); components of the mean field (Figure S3); Ramachandran distributions with different backbone geometries (Figure S4); examples of structures containing atomic overlaps resulting in high energies (Figure S5) (PDF)

## References

[ref1] Judy E.; Kishore N. A look back at the molten globule state of proteins: Thermodynamic aspects. Biophys. Rev. 2019, 11, 365–375. 10.1007/s12551-019-00527-0. 31055760 PMC6557940

[ref2] Sali A.; Shakhnovich E.; Karplus M. How does a protein fold. Nature 1994, 369, 248–251. 10.1038/369248a0. 7710478

[ref3] Haran G. How, when and why proteins collapse: The relation to folding. Curr. Opin. Struct. Biol. 2012, 22, 14–20. 10.1016/j.sbi.2011.10.005. 22104965 PMC3288525

[ref4] van der Lee R.; et al. Classification of Intrinsically Disordered Regions and Proteins. Chem. Rev. 2014, 114, 6589–6631. 10.1021/cr400525m. 24773235 PMC4095912

[ref5] Yu F.; Sukenik S. Structural Preferences Shape the Entropic Force of Disordered Protein Ensembles. J. Phys. Chem. B 2023, 127, 4235–4244. 10.1021/acs.jpcb.3c00698. 37155239 PMC10201532

[ref6] Uversky V. Natively unfolded proteins: A point where biology waits for physics. Protein Sci. 2002, 11, 739–756. 10.1110/ps.4210102. 11910019 PMC2373528

[ref7] Baxa M. C.; Lin X.; Mukinay C. D.; Chakravarthy S.; Sachleben J. R.; Antilla S.; Hartrampf N.; Riback J. A.; Gagnon I. A.; Pentelute B. L.; et al. How hydrophobicity, side chains, and salt affect the dimensions of disordered proteins. Protein Sci. 2024, 33 (5), e4986 10.1002/pro.4986. 38607226 PMC11010952

[ref8] Ramachandran G. N.; Ramakrishnan C.; Sasisekharan V. Stereochemistry of polypeptide chain configurations. J. Mol. Biol. 1963, 7, 95–99. 10.1016/S0022-2836(63)80023-6. 13990617

[ref9] Ramakrishnan C.; Ramachandran G. N. Stereochemical criteria for polypeptide and protein chain conformations. II. Allowed conformations for a pair of peptide units. Biophys. J. 1965, 5, 909–933. 10.1016/S0006-3495(65)86759-5. 5884016 PMC1367910

[ref10] Flory P. J.; Volkenstein M., Statistical Mechanics of Chain Molecues; Wiley & Sons: New York, NY, USA, 1969; pp. 699 700.

[ref11] Anfinsen C. Principles that govern folding of protein chains. Science 1973, 181, 223–230. 10.1126/science.181.4096.223. 4124164

[ref12] Levinthal C. Are there pathways for protein folding?. J. Chem. Phys. 1968, 65, 44–45. 10.1051/jcp/1968650044.

[ref13] Schweitzer-Stenner R. The relevance of short peptides for an understanding of unfolded and intrinsically disordered proteins. Phys. Chem. Chem. Phys. 2023, 25, 11908–11933. 10.1039/D3CP00483J. 37096579

[ref14] Gates Z. P.; Baxa M. C.; Yu W.; Riback J. A.; Li H.; Roux B.; Kent S. B. H.; Sosnick T. R. Perplexing cooperative folding and stability of a low-sequence complexity, polyproline 2 protein lacking a hydrophobic core. Proc. Natl. Acad. Sci. U. S. A 2017, 114, 2241–2246. 10.1073/pnas.1609579114. 28193869 PMC5338507

[ref15] Berisio R.; Vitagliano L.; Mazzarella L.; Zagari A. Crystal structure of the collagen triple helix model [(Pro-Pro-Gly)(10)](3). Protein Sci. 2002, 11, 262–270. 10.1110/ps.32602. 11790836 PMC2373432

[ref16] Hagarman A.; Measey T. J.; Mathieu D.; Schwalbe H.; Schweitzer-Stenner R. Intrinsic propensities of amino acid residues in GxG peptides inferred from amide I’ band profiles and NMR scalar coupling constants. J. Am. Chem. Soc. 2010, 132, 540–551. 10.1021/ja9058052. 20014772

[ref17] Toal S. E.; Verbaro D. J.; Schweitzer-Stenner R. Role of enthalpy-entropy compensation interactions in determining the conformational propensities of amino acid residues in unfolded peptides. J. Phys. Chem. B 2014, 118, 1309–1318. 10.1021/jp500181d. 24423055

[ref18] Shi Z. S.; Chen K.; Liu Z. G.; Ng A.; Bracken W. C.; Kallenbach N. R. Polyproline II propensities from GGXGG peptides reveal an anticorrelation with β-sheet scales. Proc. Natl. Acad. Sci. U. S. A. 2005, 102, 17964–17968. 10.1073/pnas.0507124102. 16330763 PMC1312395

[ref19] Graf J.; Nguyen P. H.; Stock G.; Schwalbe H. Structure and dynamics of the homologous series of alanine peptides: A joint molecular dynamics/NMR study. J. Am. Chem. Soc. 2007, 129, 1179–1189. 10.1021/ja0660406. 17263399

[ref20] Grdadolnik J.; Mohacek-Grosev V.; Baldwin R. L.; Avbelj F. Populations of the three major backbone conformations in 19 amino acid dipeptides. Proc. Natl. Acad. Sci. U. S. A. 2011, 108, 1794–1798. 10.1073/pnas.1017317108. 21205907 PMC3033284

[ref21] Zhang Y.; Zhou Y.; He L.; Fu Y.; Zhang W.; Hu J.; Shi Z. Hydration effects on Leu’s polyproline II population in AcLXPNH_2_. Chem. Commun. 2018, 54, 5764–5767. 10.1039/C8CC02402B. 29781018

[ref22] Ilawe N. V.; Raeber A. E.; Schweitzer-Stenner R.; Toal S. E.; Wong B. M. Assessing backbone solvation effects in the conformational propensities of amino acid residues in unfolded peptides. Phys. Chem. Chem. Phys. 2015, 17, 24917–24924. 10.1039/C5CP03646A. 26343224

[ref23] Meral D.; Toal S.; Schweitzer-Stenner R.; Urbanc B. Water-centered interpretation of intrinsic pPII propensities of amino acid residues: In vitro-driven molecular dynamics study. J. Phys. Chem. B 2015, 119, 13237–13251. 10.1021/acs.jpcb.5b06281. 26418575

[ref24] Zhang S.; Schweitzer-Stenner R.; Urbanc B. Do molecular dynamics force fields capture conformational dynamics of alanine in water?. J. Chem. Theory Comput. 2020, 16, 510–527. 10.1021/acs.jctc.9b00588. 31751129

[ref25] Yuan Y.; Wang F. Dipole Cooperativity and Polarization Frustration Determine the Secondary Structure Distribution of Short Alanine Peptides in Water. J. Phys. Chem. B 2023, 127, 3126–3138. 10.1021/acs.jpcb.2c07947. 36848625 PMC10108861

[ref26] Tran H.; Wang X.; Pappu R. Reconciling observations of sequence-specific conformational propensities with the generic polymeric behavior of denatured proteins. Biochemistry 2005, 44, 11369–11380. 10.1021/bi050196l. 16114874

[ref27] Jumper J.; Evans R.; Pritzel A.; Green T.; Figurnov M.; Ronneberger O.; Tunyasuvunakool K.; Bates R.; Žídek A.; Potapenko A.; et al. Highly accurate protein structure prediction with AlphaFold. Nature 2021, 596, 583–589. 10.1038/s41586-021-03819-2. 34265844 PMC8371605

[ref28] Ruff K. M.; Pappu R. V AlphaFold and Implications for Intrinsically Disordered Proteins. J. Mol. Biol. 2021, 433 (20), 167208 10.1016/j.jmb.2021.167208. 34418423

[ref29] Qin S.; Zhou H.-X. Predicting the Sequence-Dependent Backbone Dynamics of Intrinsically Disordered Proteins. eLife 2023, 10.7554/eLife.88958.2. PMC11524581 39475380

[ref30] Andrews B.; Guerra J.; Schweitzer-Stenner R.; Urbanc B. Do Molecular Dynamics Force Fields Accurately Model Ramachandran Distributions of Amino Acid Residues in Water?. Phys. Chem. Chem. Phys. 2022, 24, 3259–3279. 10.1039/D1CP05069A. 35048087

[ref31] Andrews B.; Schweitzer-Stenner R.; Urbanc B. Intrinsic Conformational Dynamics of Glycine and Alanine in Polarizable Molecular Dynamics Force Fields: Comparison to Spectroscopic Data. J. Phys. Chem. B 2024, 128 (25), 6217–6231. 10.1021/acs.jpcb.4c02278. 38877893 PMC11215781

[ref32] Jiang F.; Han W.; Wu Y.-D. Influence of Side Chain Conformations on Local Conformational Features of Amino Acids and Implication for Force Field Development. J. Phys. Chem. B 2010, 114, 5840–5850. 10.1021/jp909088e. 20392111

[ref33] Nerenberg P. S.; Head-Gordon T. New developments in force fields for biomolecular simulations. Curr. Opin. Struc. Biol. 2018, 49, 129–138. 10.1016/j.sbi.2018.02.002. 29477047

[ref34] Tian C.; Kasavajhala K.; Belfon K. A. A.; Raguette L.; Huang H.; Migues A. N.; Bickel J.; Wang Y.; Pincay J.; Wu Q.; Simmerling C. ff19SB: Amino-Acid-Specific Protein Backbone Parameters Trained against Quantum Mechanics Energy Surfaces in Solution. J. Chem. Theory Comput. 2020, 16, 528–552. 10.1021/acs.jctc.9b00591. 31714766 PMC13071887

[ref35] Best R. B.; Zheng W.; Mittal J. Balanced protein-water interactions improve properties of disordered proteins and non-specific protein association. J. Chem. Theory Comput. 2014, 10, 5113–5124. 10.1021/ct500569b. 25400522 PMC4230380

[ref36] Kang W.; Jiang F.; Wu Y.-D. Universal Implementation of a Residue-Specific Force Field Based on CMAP Potentials and Free Energy Decomposition. J. Chem. Theory Comput. 2018, 14, 4474–4486. 10.1021/acs.jctc.8b00285. 29906395

[ref37] Song D.; Liu H.; Luo R.; Chen H.-F. Environment-Specific Force Field for Intrinsically Disordered and Ordered Proteins. J. Chem. Inf. Model. 2020, 60, 2257–2267. 10.1021/acs.jcim.0c00059. 32227937 PMC10449432

[ref38] Zhou C. Y.; Jiang F.; Wu Y. D. Residue-specific force field based on protein coil library. RSFF2: Modification of AMBER ff99SB. J. Phys. Chem. B 2015, 119, 1035–1047. 10.1021/jp5064676. 25358113

[ref39] Jiang F.; Zhou C.-Y.; Wu Y.-D. Residue-specific force field based on the protein coil library. RSFF1: Modification of OPLS-AA/L. J. Phys. Chem. B 2014, 118, 6983–6998. 10.1021/jp5017449. 24815738

[ref40] Li S.; Elcock A. H. Residue-Specific Force Field (RSFF2) Improves the Modeling of Conformational Behavior of Peptides and Proteins. J. Phys. Chem. Lett. 2015, 6, 2127–2133. 10.1021/acs.jpclett.5b00654. 26266514 PMC4657862

[ref41] Robustelli P.; Piana S.; Shaw D. E. Developing a molecular dynamics force field for both folded and disordered protein states. Proc. Natl. Acad. Sci. U. S. A. 2018, 115, E4758–E4766 10.1073/pnas.1800690115. 29735687 PMC6003505

[ref42] Poleto M. D.; Lemkul J. A. Integration of experimental data and use of automated fitting methods in developing protein force fields. Commun. Chem. 2022, 5, 38 10.1038/s42004-022-00653-z. 35382231 PMC8979544

[ref43] Kang W.; Jiang F.; Wu Y.-D. How to strike a conformational balance in protein force fields for molecular dynamics simulations?. Wiley Interdiscip. Rev.: Comput. Mol. Sci. 2022, 12, e1578 10.1002/wcms.1578.

[ref44] Hayward S.; Kitao A.; Go N. Harmonicity and anharmonicity in protein dynamics - a normal-mode analysis and principal component analysis. Proteins: Struct., Funct., Genet. 1995, 23, 177–186. 10.1002/prot.340230207. 8592699

[ref45] Zhou A. Q.; O’Hern C. S.; Regan L. Predicting the side-chain dihedral angle distributions of nonpolar, aromatic, and polar amino acids using hard sphere models. Proteins: Struct., Funct., Bioinf. 2014, 82, 2574–2584. 10.1002/prot.24621. 24912976

[ref46] Schweitzer-Stenner R. Distribution of conformations sampled by the central amino acid residue in tripeptides inferred from amide I’ band profiles and NMR scalar coupling constants. J. Phys. Chem. B 2009, 113, 2922–2932. 10.1021/jp8087644. 19243204

[ref47] Humphrey W.; Dalke A.; Schulten K. VMD: Visual molecular dynamics. J. Mol. Graphics 1996, 14, 33–38. 10.1016/0263-7855(96)00018-5. 8744570

[ref48] Berendsen H. J. C.; van der Spoel D.; van Drunen R. GROMACS: A message-passing parallel molecular dynamics implementation. Comput. Phys. Commun. 1995, 91, 43–56. 10.1016/0010-4655(95)00042-E.

[ref49] Lindahl E.; Hess B.; van der Spoel D. GROMACS 3.0: A package for molecular simulation and trajectory analysis. J. Mol. Model. 2001, 7, 306–317. 10.1007/s008940100045.

[ref50] Van Der Spoel D.; Lindahl E.; Hess B.; Groenhof G.; Mark A. E.; Berendsen H. J. C. GROMACS: Fast, flexible, and free. J. Comput. Chem. 2005, 26, 1701–1718. 10.1002/jcc.20291. 16211538

[ref51] Hess B.; Kutzner C.; van der Spoel D.; Lindahl E. GROMACS 4: Algorithms for highly efficient, load-balanced, and scalable molecular simulation. J. Chem. Theory Comput. 2008, 4, 435–447. 10.1021/ct700301q. 26620784

[ref52] Pronk S.; Pall S.; Schulz R.; Larsson P.; Bjelkmar P.; Apostolov R.; Shirts M. R.; Smith J. C.; Kasson P. M.; van der Spoel D.; Hess B.; Lindahl E. GROMACS 4.5: A high-throughput and highly parallel open source molecular simulation toolkit. Bioinformatics 2013, 29, 845–854. 10.1093/bioinformatics/btt055. 23407358 PMC3605599

[ref53] Pall S.; Abraham M. J.; Kutzner C.; Hess B.; Lindahl E. Tackling exascale software challenges in molecular dynamics simulations with GROMACS. In Solving software challenges for exascale. 2nd International Conference on Exascale Applications and Software (EASC): 2015, 3 27.

[ref54] Abraham M. J.; Murtola T.; Schulz R.; Páll S.; Smith J. C.; Hess B.; Lindahl E. GROMACS: High performance molecular simulations through multi-level parallelism from laptops to supercomputers. SoftwareX 2015, 1–2, 19–25. 10.1016/j.softx.2015.06.001.

[ref55] Andrews B. Dihedral Rotation of Proteins (DROP). 2024; https://github.com/andrewsb8/DROP/tree/main. Accessed 1 Mar 2024.

[ref56] Huang J.; Rauscher S.; Nawrocki G.; Ran T.; Feig M.; de Groot B. L.; Grubmüller H.; MacKerell A. D. Jr. CHARMM36m: An improved force field for folded and intrinsically disordered proteins. Nat. Methods 2017, 14, 71–73. 10.1038/nmeth.4067. 27819658 PMC5199616

[ref57] Lorentz H. A. Ueber die Anwendung des Satzes vom Virial in der kinetischen Theorie der Gase. Annal. Phys. 1881, 248, 127–136. 10.1002/andp.18812480110.

[ref58] Andrews B.; Zhang S.; Schweitzer-Stenner R.; Urbanc B. Glycine in water favors the polyproline II state. Biomolecules 2020, 10, 1121 10.3390/biom10081121. 32751224 PMC7463814

[ref59] Wang A. C.; Bax A. Determination of the backbone dihedral angles ϕ in human ubiquitin from reparametrized empirical Karplus equations. J. Am. Chem. Soc. 1996, 118, 2483–2494. 10.1021/ja9535524.

[ref60] Wirmer J.; Schwalbe H. Angular dependence of ^12^coupling constants measured in J-modulated HSQCs. J. Biomol. NMR 2002, 23, 47–55. 10.1023/A:1015384805098. 12061717

[ref61] Finkelstein A. V.; Ptitsyn O. B. Protein Physics: A Course of Lectures; Academic Press: London, 2002.

[ref62] Beck D. A. C.; Alonso D. O. V.; Inoyama D.; Daggett V. The intrinsic conformational propensities of the 20 naturally occurring amino acids and reflection of these propensities in proteins. Proc. Natl. Acad. Sci. U. S. A. 2008, 105, 12259–12264. 10.1073/pnas.0706527105. 18713857 PMC2527899

[ref63] Jha A.; Colubri A.; Zaman M.; Koide S.; Sosnick T.; Freed K. Helix, sheet, and polyproline II frequencies and strong nearest neighbor effects in a restricted coil library. Biochemistry 2005, 44, 9691–9702. 10.1021/bi0474822. 16008354

[ref64] Milorey B.; Schweitzer-Stenner R.; Andrews B.; Schwalbe H.; Urbanc B. Short peptides as predictors for the structure of polyarginine sequences in disordered proteins. Biophys. J. 2021, 120, 662–676. 10.1016/j.bpj.2020.12.026. 33453267 PMC7896027

