# Supplementary material for: Amino Acid Residue-Specific Ramachandran Distributions Derived from a Simple Mean Field Potential
Source: ACS Phys Chem Au. 2024 Oct 21;4(6):707–19. doi: 10.1021/acsphyschemau.4c00064 (PMC11613349; doi:10.1021/acsphyschemau.4c00064)
Supplement: Supplementary file 1 — pg4c00064_si_001.pdf [file pg4c00064_si_001.pdf]

Supporting Information: Amino Acid Residue-Specific Ramachandran Distributions Derived from a Simple Mean Field Potential

Brian Andrews*

Department of Physics, Bryn Mawr College, Bryn Mawr, PA 19010, USA

E-mail: bandrews1@brynmawr.edu

1 Supporting Tables

Table S1: Minimum contact distance between atom type pairs. Values taken from second column of Table 1 in Ramachandran.[S1]

Atom Pairs	Minimum Distanced ($\AA$)
C-C	3.00
C-O	2.70
C-N	2.80
C-H	2.20
O-O	2.70
O-N	2.60
O-H	2.20
N-N	2.60
N-H	2.20
H-H	1.90

Table S2: Parameters used for each atom for calculation of the intrapeptide Lennard-Jones energies taken from CHARMM36m.[S2]

Atom	Atom Type	ϵ [kJ/mol]	σ [$\AA$]
C	Carbonyl C (peptide backbone)	0.46024	3.56359
O	Carbonyl Oxygen	0.50208	3.02905
N	Amide Nitrogen	0.83680	3.29632
H	Nonpolar H	0.09204	2.35197

Table S3: Mesostate populations derived from the Mean Field (this work) and Gaussian model and MD-derived populations from Ref.[S3] (pβ values not reported). Values for the Mean Field are reported for Ramachandran distributions generated using the following λ values: 1.0 for alanine, 0.90 for valine, 0.90 for leucine, and 0.75 for isoleucine.

Method	pPII	βt	aβ	pβ	α
Alanine					
Mean Field	0.69	0.24	0.02	0.00	$<$ 0.01
Gaussian Model[a]	0.59	0.16	0.02	-	0.02
CHARMM36m[a]	0.55	0.09	0.12	-	0.06
Valine					
Mean Field	0.34	0.28	0.36	0.00	$<$ 0.01
Gaussian Model[a]	0.30	0.40	0.02	-	0.02
CHARMM36m[a]	0.55	0.11	0.05	-	0.06
Leucine					
Mean Field	0.85	0.10	0.02	0.01	0.02
Gaussian Model[a]	0.43	0.18	0.01	-	0.00
CHARMM36m[a]	0.51	0.13	0.05	-	0.07
Isoleucine					
Mean Field	0.74	0.03	0.00	0.23	0.00
Gaussian Model[a]	0.28	0.10	0.01	-	0.06
CHARMM36m[a]	0.49	0.12	0.06	-	0.09

[a] Data taken from Andrews *et al.*[S3]

Table S4: Karplus parameters used to calculate the NMR J-coupling constants in this work.

Coupling Constant	A	B	C	θ	Source
$^3J(H^N, H^{C_\alpha})$	6.98	-1.38	1.72	-60	Wang and Bax[S4]
$^1J(N, C_\alpha)$	2.84	-1.21	8.64	0	Wirmer and Schwalbe[S5]

Table S5: NMR Coupling constants calculated in the study. Values for the Mean Field (this work) are reported for Ramachandran distributions generated using the following λ values: 1.0 for alanine, 0.90 for valine, 0.90 for leucine, and 0.75 for isoleucine.

Method	$^3J(H^N, H^{C_\alpha})$	$^1J(N, C_\alpha)$
Alanine		
Experimental[a]	6.11 ± 0.02	11.28 ± 0.07
Mean Field	7.39	9.55
Gaussian Model[a]	6.00	11.39
CHARMM36m[a]	6.37	11.21
Valine		
Experimental[a]	7.46 ± 0.02	11.24 ± 0.07
Mean Field	8.39	10.35
Gaussian Model[a]	7.43	11.50
CHARMM36m[a]	6.58	10.98
Leucine		
Experimental[a]	6.78 ± 0.01	10.96 ± 0.10
Mean Field	6.48	9.47
Gaussian Model[a]	6.72	11.11
CHARMM36m[a]	6.61	11.02
Isoleucine		
Experimental[a]	7.47 ± 0.01	10.88 ± 0.07
Mean Field	6.82	9.50
Gaussian Model[a]	7.33	10.97
CHARMM36m[a]	6.71	10.86

[a] Data taken from Andrews *et al.*[S3]

2 Supporting Figures

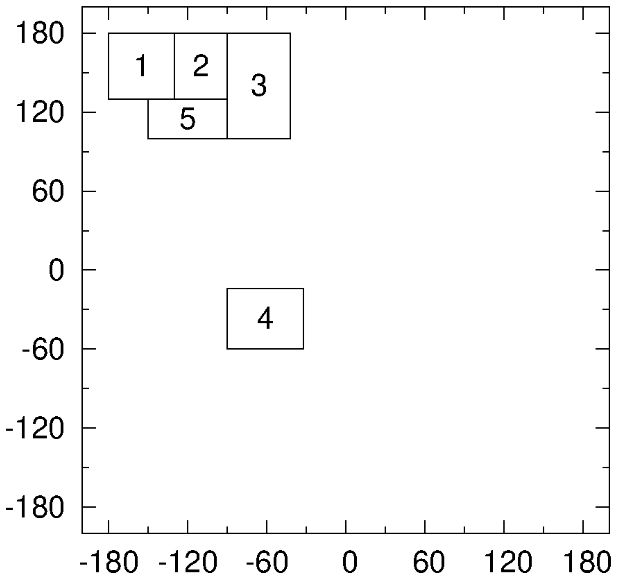

Figure S1: The regions of Ramachandran space as defined in *Methods*: (1) aβ, (2) βt, (3) pPII, (4) α, (5) pβ.

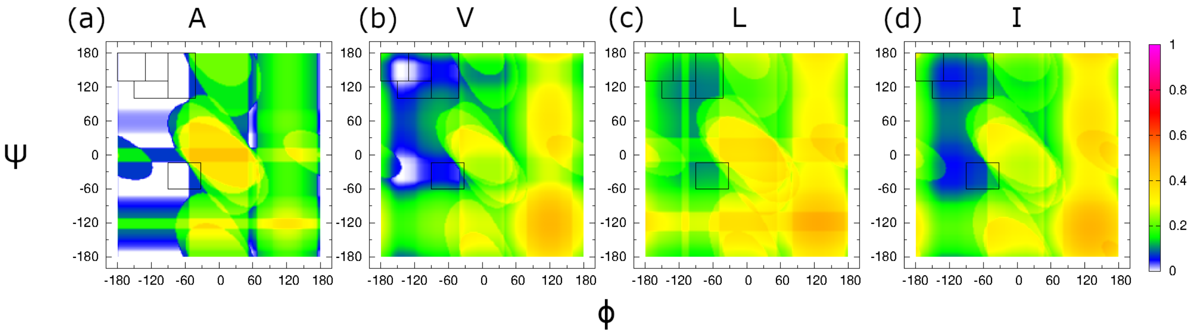

Figure S2: (a-d) Average number of atomic overlaps, normalized by number of atoms in the mimetic structure, in Ramachandran space for alanine, valine, leucine, and isoleucine amino acid mimetics.

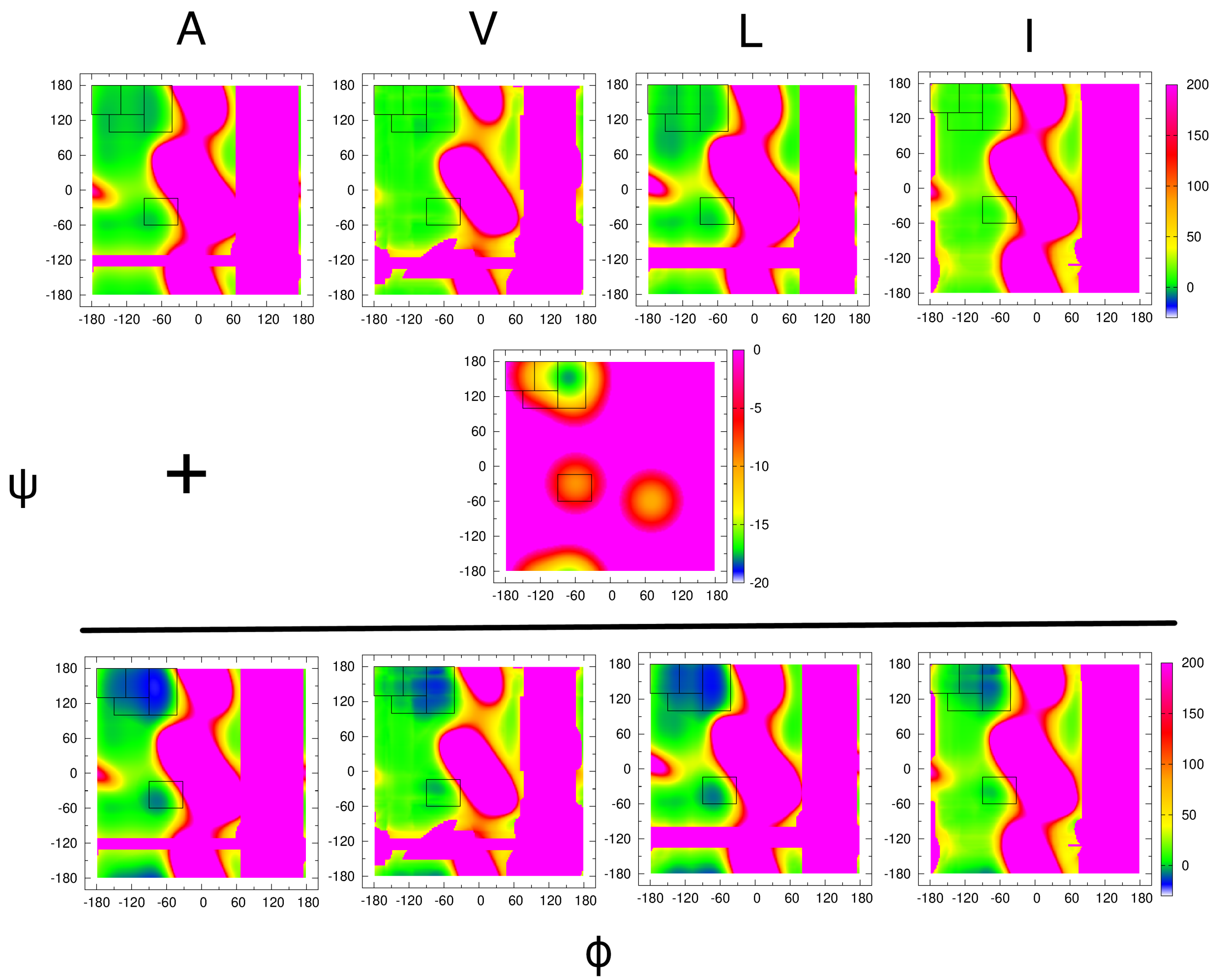

Figure S3: Example of contributions to the mean fields by (top row) the average LJ energies for each backbone dihedral angle, (center row) the backbone-water energy contribution, and (bottom row) the sum of the two components for $\lambda = 1$ for amino acid residues alanine, valine, leucine, and isoleucine. The z axis has units of kJ/mol.

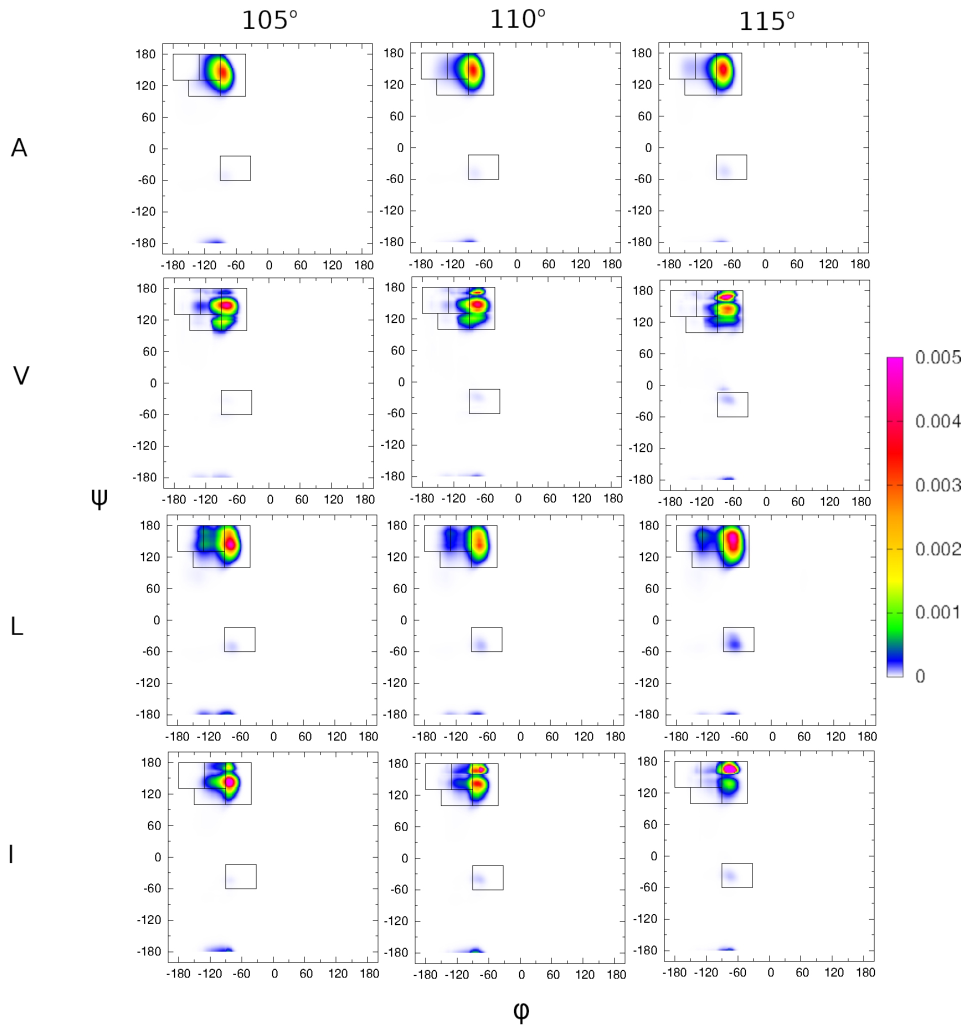

Figure S4: Ramachandran distributions produced for three different backbone N-C_α-C' angles for each amino acid residue mimetic considered in this work for $\lambda = 1.0$. The central column is reproduced from the first row of Fig. 3 in the main manuscript. For V, L, and I, higher backbone angles lead to a positive shift of the minimum in the energy landscape to higher ϕ and ψ.

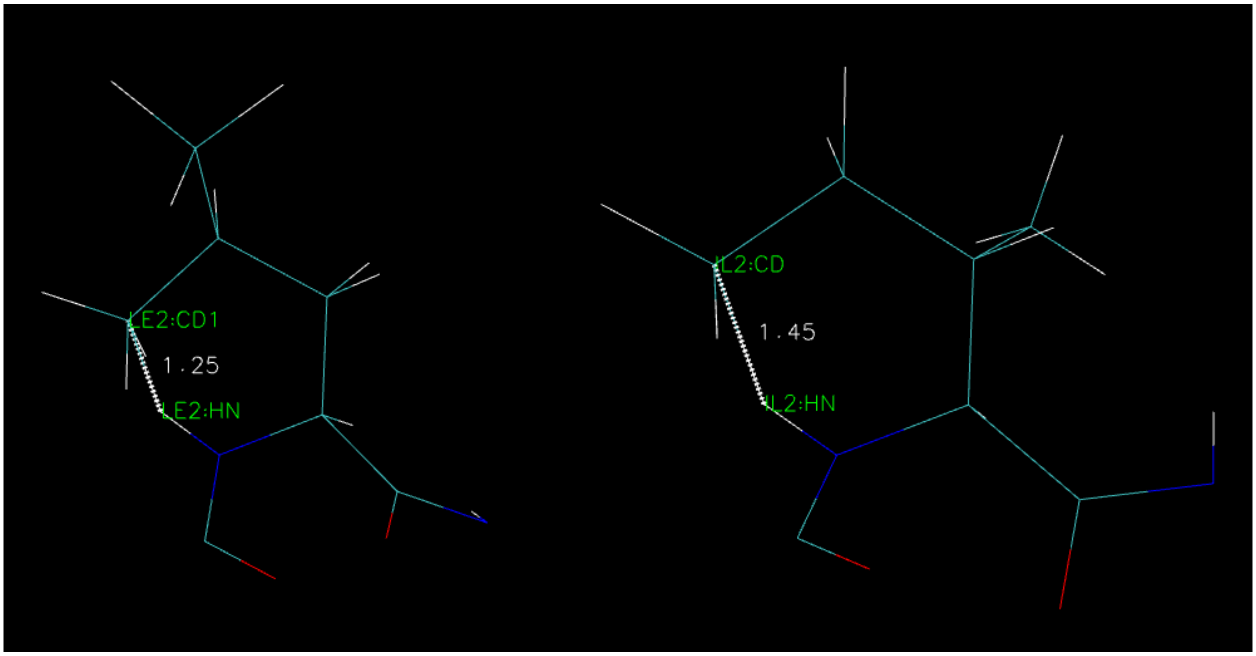

Figure S5: Example structures of the (left) leucine mimetic and (right) isoleucine mimetic where both side chain dihedral angles are set to 0°. As a result the amide hydrogen is 1.25 $\AA$ and 1.45 $\AA$ from the C_D atom of the side chains of leucine and isolecuine, respectively. This close proximity results in very large LJ energy values and the cutoff values utilized in this study prohibit their influence on the resultant mean field.

References

(S1) Ramachandran, G. N.; Ramachandran, C.; Sasisekharan, V. Stereochemistry of polypeptide chain configurations. *J. Mol. Biol.* **1963**, *7*, 95.

(S2) Huang, J.; Rauscher, S.; Nawrocki, G.; Ran, T.; Feig, M.; de Groot, B. L.; Grubmüller, H.; MacKerell, A. D., Jr. CHARMM36m: an improved force field for folded and intrinsically disordered proteins. *Nature Methods* **2017**, *14*, 71–73.

(S3) Andrews, B.; Guerra, J.; Schweitzer-Stenner, R.; Urbanc, B. Do Molecular Dynamics Force Fields Accurately Model Ramachandran Distributions of Amino Acid Residues in Water? *Phys. Chem. Chem. Phys.* **2022**, *24*, 3259–3279.

(S4) Wang, A. C.; Bax, A. Determination of the backbone dihedral angles ϕ in human ubiquitin from reparametrized empirical Karplus equations. *J. Am. Chem. Soc.* **1996**, *118*, 2483–2494.

(S5) Wirmer, J.; Schwalbe, H. Angular dependence of ${}^1J(N_i, C_{\alpha i})$ and ${}^2J(N_i, C_{\alpha(i-1)})$ coupling constants measured in J-modulated HSQCs. *J. Biomol. NMR* **2002**, *23*, 47–55.
